# Sizing up competition with strigolactones: the case of pea plants

**DOI:** 10.1080/15592324.2025.2506556

**Published:** 2025-05-19

**Authors:** Bianca Bonato, Tom Bennett, Emanuele Cannizzo, Sara Avesani, Silvia Guerra, Umberto Castiello

**Affiliations:** a Department of General Psychology, University of Padova, Padova, Italy; b Faculty of Biological Science, University of Leeds, Leeds, UK

**Keywords:** Strigolactones, plant behavior, kinematics, competition, pea plant, climbing plant, *Pisum sativum*

## Abstract

Plants, though sessile, can detect and respond to their neighbors through chemical signals such as strigolactones (SLs). We investigated how SL synthesis and perception affect the climbing behavior of *Pisum sativum* by analyzing wild-type plants and two SL-related mutants—*rms1–1* (SL-deficient) and *rms3–1* (SL-insensitive) – grown either alone or paired with a plant of a different genotype but of the same genetic background. Using 3D kinematic analysis, we quantified the circumnutation and attachment dynamics. Our results show that social context significantly modulated climbing behavior. *rms1–1* mutants, although unable to grasp the support, showed increased movement velocity in social conditions, suggesting enhanced exploratory behavior. In contrast, *rms3–1* mutants exhibited slower, disoriented movements when paired, indicating impaired neighbor perception. Wild-type plants successfully grasped the support in all conditions but altered their behavior socially, increasing movement velocity with a more careful approaching phase. These results show that SL-mediated signaling, through both emission and perception, shapes context-dependent climbing strategies in pea plants.

## 1. Introduction

Plants possess a limited ability to select their neighbors, yet they engage in a wide range of social interactions that enhance their survival across diverse ecological contexts. By perceiving their neighbors, plants can anticipate potential interactions and modify their behavior to optimize long-term benefits.^1^ Among the social attitudes exhibited by plants competition for above- and below-ground resources is often intense and has important consequences.^2^

Chemical signaling plays a crucial role in the detection of neighboring plants, and it is also integral for competitive interactions, where plants release chemical substances to engage in chemical defense against their competitors.^3^ In essence, plants appear to make decisions that are contingent on varying ecological conditions, elaborating the information from the context and the neighbors’ identity and then implementing appropriate flexible behavioral responses.

This process likely involves a complex array of chemicals including primary and secondary metabolites,^4^ light patterns,^5^ mechanosensing stimuli^6^ and chemical exudates by the roots (i.e, root exudates^3,7–9^). Among these, strigolactones (SLs), a class of plant hormones, play an important role in mediating plant responses by acting as plant–plant signaling molecules, facilitating interplant communication and neighbor detection.^10–12^

These aspects have been tested by Wheeldon et al.^11^ using mixed cultures of pea *ramosus* mutants that disrupt SL synthesis and signaling. These authors inferred that SL-deficient *rms1–1* mutants should be able to detect neighbors but would be less detectable to their neighbors since they cannot exude SLs in the growth substrate. Conversely, SL-insensitive *rms3–1* mutants should be less receptive to other plants, but still detectable to *rms1–1* and wild-type neighbors since they continue to exude SLs. Relative to *rms1–1* plants grown in monoculture, a solitary *rms1–1* plant surrounded by *rms3–1* plants exhibited substantially reduced shoot growth, while the *rms3–1* plants all grew more strongly than in monoculture. The suggestion was that this growth boost was due to reduced competition from the *rms1–1* neighbor.

In the present study, we capitalize on this evidence by investigating the ascent and attachment behavior of wild-type pea plants and *rms1–1* and *rms3–1* mutants in a competitive context using 3D motion analysis.^13,14^ Kinematical analysis has proved to be particularly effective in establishing differences in the ascent and attachment behavior of *rms1–1* and *rms3–1* mutants with respect to wild-type plants^15^ and to characterize the circumnutative pattern of two pea plants competing for accessing a potential support.^13^ In the latter study, distinct kinematic patterns for the two competing plants were observed. The “winning” plant, the one grasping the support, was faster, and the spatial trajectories were very soon oriented toward the support. In contrast, the “losing” plant exhibited a much slower movement, and once the plant ‘recognized’ the winning status of the other plant, it redirected its search for alternative supports.

With this in mind, we test the ascent and attachment behavior of *rms1–1* mutants potted together with a wild-type sharing the same genetic background (i.e., L77) and *rms3–1* mutants potted together with a wild-type sharing the same Torsdag background line. If plants exchange and re-absorb the amount of SLs secreted in the soil, then diverse combinations of wild type and mutant, that either do not synthesize SLs or do not perceive SLs, should determine different outcomes of social interaction played out in the kinematics of ascent and attach behavior. We also tested the behavior of mutant plants and wild-types for each genotype individually. This aspect is vital to discern between the two main hypotheses driving the study. First, we hypothesized that these genotypes would behave differently between social and solitary conditions. Second, we hypothesized that there are behavioral and morphological differences across different genotypes when engaged in social interactions.

## 2. Material and methods

### 2.1. Subjects

Eight *Pisum Sativum* L. wildtypes with genotype Torsdag, eight *Pisum Sativum* L. wildtypes with genotype L77, eight rms3–1 (Torsdag background) and eight rms1–1 (L77 background) *Pisum Sativum* L. mutant plants^16,17^ were tested. Four plants for each individual condition and four couple (i.e., two plants per couple) for the social conditions.

**Figure 1.**
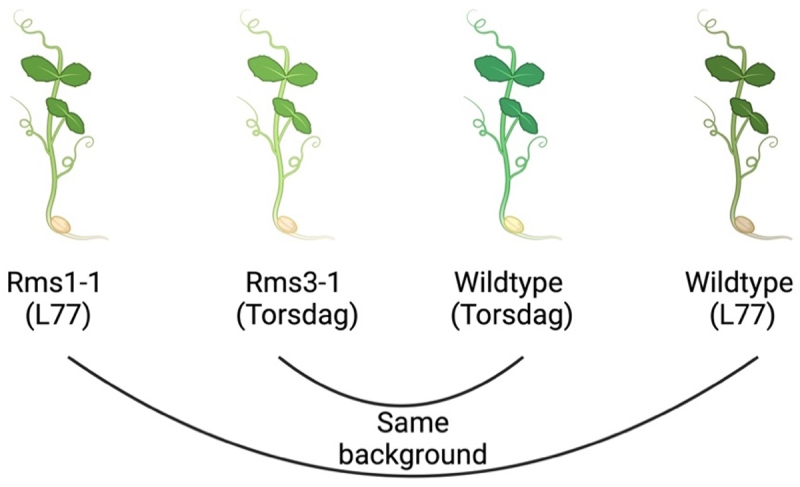
Graphical representation of the subjects with different genotypes selected for the study.

### 2.2. Support

The support was a wooden pole 60 cm in height (6 cm underground and 54 cm above ground) and 0.5 cm in diameter.

### 2.3. Germination and growth conditions

Seeds were germinated over a period of 5 days on strips of filter paper moistened with water, spaced 1.5 cm apart from each other and positioned 0.5 cm from the top edge of the strip. The seeds were placed so that both the hilum and micropyle faced downward. Once germinated, healthy seedlings at the same stage of development were selected and transplanted into plastic pots.

For the individual condition, a pot measuring 20 cm in both diameter and height was used. In contrast, the social condition used a pot with a diameter of 30 cm and a height of 14 cm. All pots were filled with silica sand (type 16SS, grain size 0.8/1.2 mm, weight 1.4). At the start of each experimental condition, both pot types were irrigated and supplied with a half-strength nutrient solution (Murashige and Skoog Basal Salt Micronutrient Solution; 10× concentrate, liquid form, tested for plant cell culture; SIGMA Life Science). The combination of soil volume and nutrient solution was sufficient to support appropriate growing conditions for plants under both individual and social setups.

Watering was carried out three times per week. Each pot was placed within a growth chamber (Cultibox SG combi, dimensions 80 × 80 × 160 cm), allowing cultivation under controlled environmental parameters. Air temperature in the chamber was maintained at 26 °C, fluctuating slightly within a range of 24 °C to 26 °C across the light – dark cycle. The ventilation system included an extractor fan with a thermostat control (TT125; 125 mm diameter; max flow rate 280 m^3^/h) and an intake fan (Blauberg Tubo 100; 102 m^3^/h). Together, these fans ensured a stable airflow within the chamber, with an average air turnover time of 60 s. The fan was carefully positioned so that air currents would not interfere with the natural movements of the plants.

Plants were exposed to a daily photoperiod of 11.25 h (from 5:45 a.m. to 5:00 p.m.) using a cool white LED lighting system (V-TAC innovative LED lighting, VT-911-100W, Des Moines, IA, USA, or 100W Samsung UFO, 145 lm/W – LIFUD), mounted 57 cm above each seedling. The Photosynthetic Photon Flux Density (PPFD) measured at seedling height under the lamp was 350 μmol photons/m^2^/s, using a quantum sensor (LI-190 R, Lincoln, Nebraska, USA). Reflective Mylar® film lining the chamber walls improved the evenness of light distribution.

### 2.4. Experimental conditions

**Figure 2.**
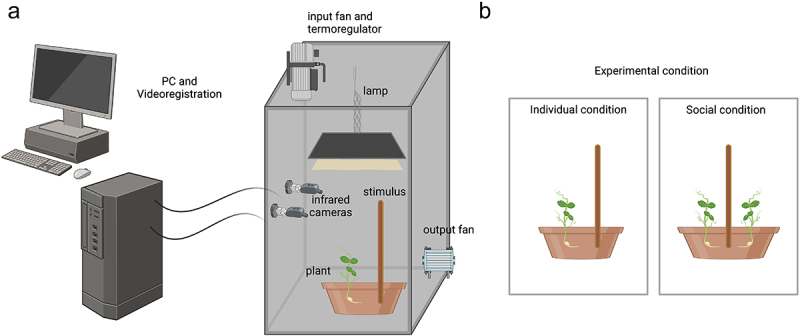
Graphical representation of the experimental set up. In panel (a) is represented the growth chamber with all the facilities; in panel (b) is represented the control condition with a solitary plant growing in the pot with the support positioned nearby (left panel) and two plants potted together and equidistant from the support positioned in the middle (right panel).

In order to study the influence of the deficiency in SLs production or perception in plants situated in a social context, we capitalized on a paradigm that was successful in identifying kinematical characteristics of social behavior.^13,18^ We implemented two experimental conditions: (i) a social condition in which an SL-insensitive *rms3–1* mutant plant, lacking a functional SL receptor, was combined with a wild-type of the same genetic background (i.e., Torsdag). This mutant should be insensitive to the presence of neighbors early in the life cycle^11^; (ii) a social condition with a *rms1–1* mutant plant that does not synthesize SLs^19^ combined with a wild type of the same genetic background (i.e., L77), capable of SLs production and perception. A series of individual conditions serving as controls for each genotype (*rms1–1, rms3–1, wild type L77* and *wild-type Torsdag)* were also considered. A single plant was potted in the presence of a potential support (i.e. stimulus) positioned at 10 cm from the plant (Figure 2b).

### 2.5. Dependent measures

The dependent variables were specifically selected to evaluate our experimental hypothesis, drawing on prior kinematic research on approach-to-grasp behavior in pea plants under social conditions^13,18^. These variables included (i) the movement paths traced by the tendril tip as 3D trajectory (Figure 2), (ii) the frequency of circumnutation events, (iii) the total time spent by the tendril performing circumnutations, (iv) the average velocity amplitude of the tendril during these movements, and (v) the distance from the plant’s circumnutation center of gravity to the support. In addition, one morphological parameter related to the below-ground portion of the plant was assessed: (vi) the root surface area (RSA). RSA is a critical and responsive indicator of plant competitive interactions, particularly belowground. It directly reflects a plant’s capacity to explore soil volume, access water and nutrients, and interact with neighboring root systems. In competitive environments, plants often alter root morphology and distribution to optimize resource acquisition, increasing RSA.^20^

### 2.6. Kinematical recording

In each growth chamber, a pair of RGB-infrared cameras (IP 2.1 Mpx, varifocal IR 1080P, outdoor-ready) was installed at a height of 110 cm above the ground, with a horizontal spacing of 45 cm between them, to capture stereo imagery of the plants. These cameras were connected via Ethernet to a 10-port wireless router (D-Link DSR-250N), which in turn communicated with a PC over Wi-Fi. Image acquisition and storage were managed using CamRecorder software (Ab.Acus s.r.l., Milan, Italy).

To enhance the visibility of specific plant structures, such as the tendrils, against the background, black felt velvet was attached to select sections of the chamber walls to increase contrast. Each camera’s internal and external parameters, along with lens distortion characteristics, were calibrated using the Camera Calibrator App in MATLAB. Depth information was derived from single-camera images by capturing 20 photographs of a chessboard pattern (square size: 18 mm, 10 columns × 7 rows) at various angles and distances under diffuse, natural lighting.

For stereo calibration, the same chessboard was positioned centrally within the chamber, and both cameras simultaneously captured images to compute the stereo calibration parameters. In line with the experimental design, cameras recorded a frame every 3 min (0.0056 hz), synchronously capturing plant development over time. The analysis focused on tendrils emerging from the targeted node. When tendrils made contact with the support structure, the coiling leaf was analyzed. The first frame used for analysis was the one in which the selected tendril became visible at the apex. Movement was considered complete in the frame where the tendril began wrapping around the support, ceased movement, or fell.

A custom software tool (SPROUT, Ab.Acus s.r.l., Milan, Italy),^14^ developed using MATLAB and Python, was employed to label anatomical landmarks using visual markers and to track these markers frame by frame using the stereo images. Markers were manually placed on the tip of the tendril after recording. Initial tracking of the tendril’s motion over time was conducted automatically using the Kanade-Lucas-Tomasi (KLT) algorithm on distortion-corrected images. Each tracked marker’s position was reviewed manually by an experimenter to ensure accuracy. The 3D path of each marker was reconstructed by triangulating the 2D trajectories obtained from the stereo camera pair.

### 2.7. Morphological measurements

At the conclusion of the experiment, plants were carefully removed from their planting units to assess the root surface area (RSA), a key metric for evaluating the plant’s ability to absorb water and nutrients.^20^ To conduct this analysis, root systems were scanned using a mobile scanning application (CamScanner, INTSIG Information Co., Ltd, Shanghai, China; Version 5.51.0). High-resolution images were captured with a smartphone (iPhone X), which provided clear contrast, sharp detail, and minimal distortion, ensuring reliable visual data.^21^

The root images were then converted into 8-bit grayscale format and imported into ImageJ software for processing. A measurement scale was applied to ensure accurate quantification, and a consistent threshold setting was used across all images to maintain uniformity in the analysis. By measuring RSA, we can detect shifts in root proliferation and spatial occupation, which are sensitive to competitive pressure. Moreover, the root surface area integrates both root length and thickness, providing a comprehensive measure serving as a meaningful dependent variable for assessing the outcome and intensity of plant competition.

### 2.8. Statistical analysis

Statistical analyses were carried out using both Bayesian inference and classical frequentist methods. Separate approaches were applied to the kinematic and morphological datasets due to differences in data distribution – kinematic data did not follow a normal distribution, whereas morphological data did. Prior to conducting any formal analyses, the Shapiro–Wilk test was used to assess the normality of the data.

For the kinematic variables, Mann–Whitney U test was employed, as the distribution of these measures did not meet the assumptions of normality. Descriptive statistics were calculated, including the median, interquartile range (IQR), full range, and the 25th, 50th, and 75th percentiles. The Mann–Whitney U test, a non-parametric alternative to the t-test, was selected because it does not rely on the assumption of normally distributed data. In this test, the W statistic is derived from the rank sums of one sample, adjusting for the smallest value.

Regarding the morphological parameters, a one-way ANOVA was used to compare group means, followed by Tukey’s post-hoc test to identify specific pairwise differences. This method assumes that the groups being compared have equal sample sizes and variances, making it suitable for the morphological data set.

All statistical procedures were conducted using JASP software^22^ integrated within the R programming environment,^23^ with supporting packages listed at: https://jaspstats.org/rpackage-list/. The null hypothesis stated that no significant differences existed between experimental groups in terms of either movement patterns or root surface area, while the alternative hypothesis proposed that differences were present.

## 3. Results

### 3.1. Qualitative observations

When comparing the different genotypes, clear morphological differences in the aerial parts of the plants are immediately noticeable. In particular, *rms1–1* mutant plants exhibit a reduced stem length compared to both wild-type plants and other mutants. Although they reach a height that would theoretically allow them to perceive and coil around a support placed at a distance of 10 cm, they fail to do so. Similarly, *rms3–1* mutants do not show significant differences in stem length or growth compared to wild-type plants, yet also do not interact with the support. The wild-type lines L77 and Torsdag display similar morphological features and stem lengths. These observations can be directly assessed from the video material provided in the supplementary section.

#### 3.1.1. Individual conditions

**Figure 3.**
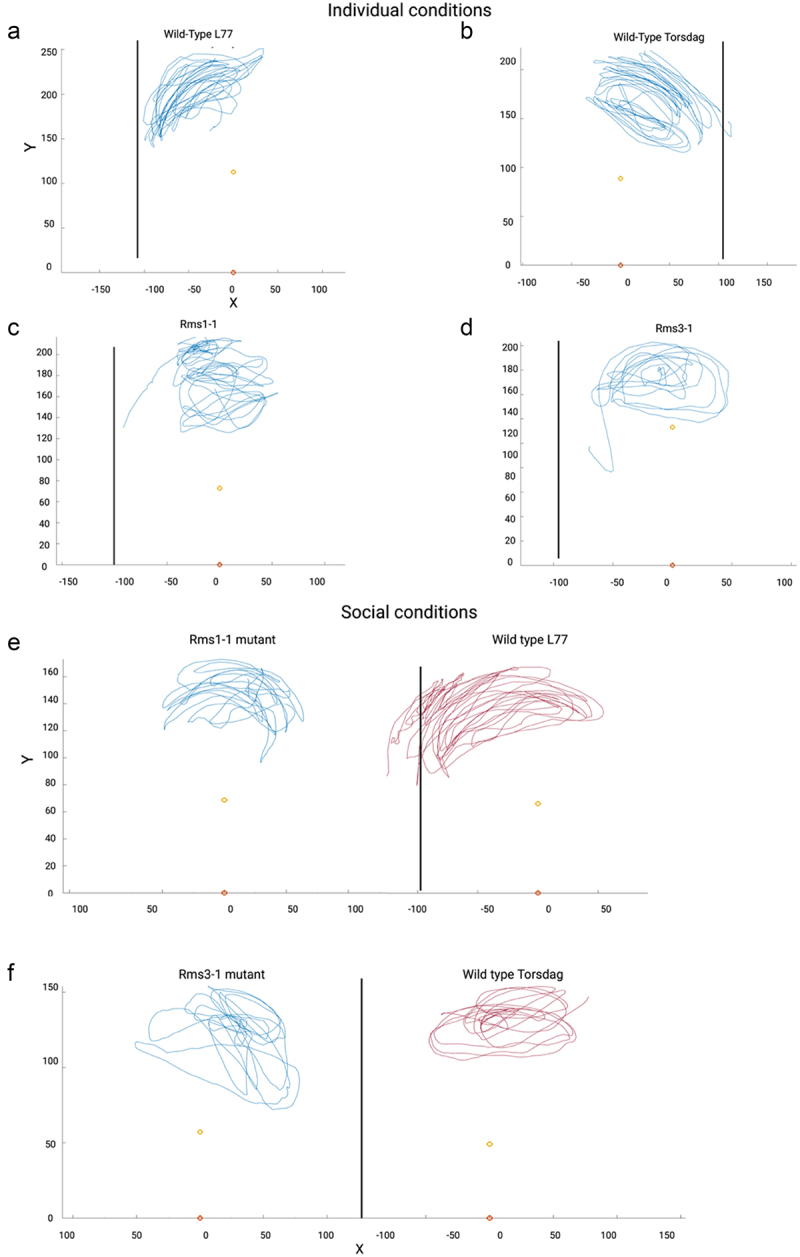
Illustration of the trajectory of the tendril for representative plants for each genotype and condition. Black vertical line represents the support; yellow and orange dots represent the internode and the origin of the plant, respectively; blue and red lines represent the trajectories of the tendrils.

When looking at the trajectories of the tendrils concerned with the individual conditions (Figure 3a-d), a circumunutative movement was observed for all the considered genotypes. Whereas the clasping of the support is evident for the wild types (Figure 3a,b), no occurrence of such behavior was observed for the mutant plants (Figure 3c,d).

#### 3.1.2. Social conditions

For the social conditions, we observed a circumnutative movement for all the considered genotypes. Despite a general inclination of all plants to move toward the support, only the wildtype (L77) potted together with the *rms1–1* mutant (Figure 3e) seems able to grasp the support. When considering the wildtype Torsdag potted together with the *rms3–1* mutant no evidence of support clasping was noticed.

### 3.2. Kinematic results

From the descriptive statistics of the kinematic analysis, it is immediately recognizable that there is a substantial difference depending on both the genotypes and social conditions (Table 1; see supplementary videos). These differences are further discussed in the next sections.

**Table 1.** Descriptive statistics for all the conditions and genotypes.

Dependent variable	Condition	Genotype	Median	IQR	Range	25th percentile	50th percentile	75th percentile
Number of circumnutations	Individual	WT L77	5.500	7.000	15.000	3.000	5.500	10.000
		WT Tor.	9.000	9.000	18.000	5.000	9.000	14.000
		RMS1	9.000	9.000	22.000	5.000	9.000	14.000
		RMS3	7.000	7.000	19.000	4.000	7.000	11.000
	Social	WT L77	10.000	9.500	24.000	5.000	10.000	14.500
		WT Tor.	8.000	7.750	16.000	4.000	8.000	11.750
		RMS1	8.000	8.500	24.000	4.000	8.000	12.500
		RMS3	6.000	6.000	12.000	3.000	6.000	9.000
Duration of circumnutations (min)	Individual	WT L77	67.500	18.750	66.000	62.250	67.500	81.000
		WT Tor.	99.000	21.000	96.000	87.000	99.000	108.000
		RMS1	108.000	36.000	168.000	96.000	108.000	132.000
		RMS3	96.000	54.750	255.000	78.000	96.000	132.750
	Social	WT L77	72.000	18.000	69.000	66.000	72.000	84.000
		WT Tor.	117.000	98.250	192.000	72.000	117.000	170.250
		RMS1	99.000	33.000	99.000	78.000	99.000	111.000
		RMS3	168.000	72.000	201.000	130.500	168.000	202.500
Amplitude of mean velocity (mm/min)	Individual	WT L77	3.466	1.978	4.913	2.424	3.466	4.403
		WT Tor.	2.646	1.006	2.904	1.991	2.646	2.996
		RMS1	1.324	1.459	5.045	0.708	1.324	2.167
		RMS3	2.042	2.059	4.505	0.773	2.042	2.832
	Social	WT L77	3.173	1.791	4.992	2.065	3.173	3.856
		WT Tor.	0.997	0.523	1.443	0.759	0.997	1.282
		RMS1	1.920	0.727	3.702	1.553	1.920	2.280
		RMS3	0.924	0.790	1.656	0.577	0.924	1.367
Distance from the support (mm)	Individual	WT L77	81.527	18.236	48.182	71.586	81.527	89.822
		WT Tor.	103.536	39.405	125.409	94.324	103.536	133.729
		RMS1	83.989	23.530	307.472	76.297	83.989	99.827
		RMS3	69.539	19.138	76.994	60.596	69.539	79.734
	Social	WT L77	68.823	23.689	77.434	53.517	68.823	77.206
		WT Tor.	124.289	100.991	148.687	54.530	124.289	155.520
		RMS1	94.700	52.127	118.369	81.283	94.700	133.411
		RMS3	92.948	21.387	73.915	86.152	92.948	107.539

### 3.3. Comparing individual and social behavior for each genotype

#### 3.3.1. Wild-type L77

The results indicate that these plants behave similarly in both the individual and the social conditions for both the duration of circumnutation (BF_10_ = 1.311, W = 1156.500, Rhat = 1.006) and the amplitude of mean velocity (BF_10_ = 0.242, W = 1702.000, Rhat = 1.019; see Supplementary Table S1). However, they differ when considering the number of circumnutation (BF_10_ = 9.432, W = 971.500, Rhat = 1.013; see Supplementary Table S1) with a higher amount of circumnutations in the social condition (see Table 1), and also when considering the distance of the plant from the support during the movement time (BF_10_ = 403.677, W = 2312.000, Rhat = 1.051; see Supplementary Table S1), which is reduced in the social condition (Table 1). Nevertheless, wild-type L77 grasps the support for both the individual and the social condition.

#### 3.3.2. Wild-type Torsdag

When considering these plants no differences between the individual and the social condition for the number of circumnutation (BF_10_ = 0.670, W = 2358.500, Rhat = 1.000; see Supplementary Table S2), the duration of circumnutation (BF_10_ = 0.856, W = 1652.500, Rhat = 1.013; see Supplementary Table S2) and the distance of the plant from the support (BF_10_ = 0.224, W = 1447.000, Rhat = 1.022; Supplementary Table S2) were found. A difference across conditions is evident for the amplitude of mean velocity (BF_10_ = 2.633 × 10^+7^, W = 4051.000, Rhat = 1.007; see Supplementary Table S2), which is lower for the social than the individual condition (Table 1).

#### 3.3.3. rms1–1

For these plants, which are not able to synthesize SLs we observed a difference between the individual and the social condition for the duration of circumnutation (BF_10_ = 35.598, W = 4949.500, Rhat = 1.024; see Supplementary Table S3), which is longer in the individual condition (Table 1), and the amplitude of mean velocity (BF_10_ = 16.958, W = 2416.000, Rhat = 1.058; Supplementary Table S3), which is lower for the individual condition (Table 1). The number of circumnutations (BF_10_ = 0.179, W = 3969.000, Rhat = 1.005; see Supplementary Table S3) and the distance of the plant from the support (BF_10_ = 0.336, W = 2875.000, Rhat = 1.059; see Supplementary Table S3) do not vary across conditions.

#### 3.3.4. rms3–1

These plants, which are not able to detect SLs. exhibit a similar number of circumnutations for the individual and the social condition (BF_10_ = 0.371, W = 2442.000, Rhat = 1.008; see Supplementary Table S4). A difference across conditions emerges for the duration of circumnutation (BF_10_ = 43.199, W = 1123.500, Rhat = 1.029; see Supplementary Table S4), with a longer duration in the social condition (Table 1), the amplitude of mean velocity (BF_10_ = 27.946, W = 3105.000, Rhat = 1.029; Supplementary Table S4), which is lower in the social condition (Table 1) and the distance from the support (BF_10_ = 3847.743, W = 618.000, Rhat = 1.106; Supplementary Table S4).

### 3.4. Social conditions

#### 3.4.1. Comparing rms1–1 with L77 wildtype

**Figure 4.**
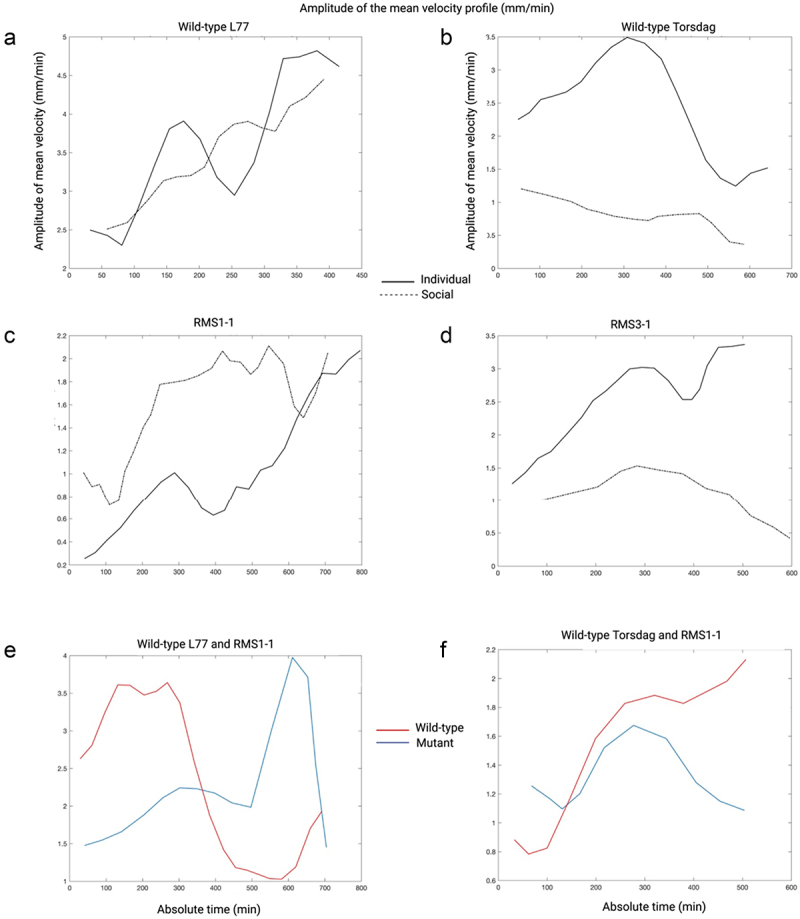
Velocity profiles for representative plants for each genotype and combination. Panel (a) represents the amplitude of the mean velocity of the tendril for the Wild-type L77 genotype for the individual and the social condition; panel (b) represents the mean velocity of the tendril for the individual and the social condition of Wil-type Torsdag; panel (c) represents the amplitude of the mean velocity of the tendril for *rms1–1* mutant; panel (d) represents the amplitude of the mean velocity of the tendril for *rms3–1* mutant; panel (e) represents the amplitude of the mean velocity for each plant within the combination Wild-type L77 and Rms1–1 mutant and panel (F) represents the amplitude of the mean velocity for each plant within the combination Wild-type Torsdag with Rms3–1 mutant.

When comparing the *rms1–1* mutant with the wild-type L77, the *rms1–1* mutant exhibited a lower number of circumnutations. Regarding the temporal aspects of the movement, the duration of circumnutation was longer for the mutant compared to the wild-type, and the amplitude of mean velocity was lower for the mutant than for the wild type (Figure 4a and Table 2). The highest amplitude of mean velocity was observed for the wild-type L77, which was the only genotype capable of completing the grasping phase (Figure 4a and Table 2). Considering the spatial aspects of the movement, the distance from the circumnutation’s center of gravity to the support was greater for the mutant than for the wild-type L77 (Table 2).

**Table 2.** Independent samples Mann-Whitney T-Test between RMS1–1 and WT L77.

	W	p
Number of circumnutations	9308.000	0.012
Duration of circumnutations (min)	5265.500	<.001
Amplitude of mean velocity (mm/min)	11273.000	<.001
Distance from the support (mm)	3666.000	<.001

Mann-Whitney U test.

**Table 3.** Independent samples Mann-Whitney T-Test between RMS3–1 and WT torsdag.

	W	p
Number of circumnutations	4116.000	0.053
Duration of circumnutations (min)	6124.000	0.002
Amplitude of mean velocity (mm/min)	4233.000	0.102
Distance from the support (mm)	3718.000	0.195

Mann-Whitney U test.

#### 3.4.2. Comparing rms3–1 with Torsdag wildtype

When examining the *rms3–1* mutant in comparison to the wild-type Torsdag both executed the same number of circumnutations (see Table 3). Additionally, the movement duration and the amplitude of mean velocity did not differ across genotypes (see Table 3).

**Table 4.** Descriptive statistics for the root surface area (cm^2^) of all the genotypes in both individual and social conditions.

Conditions	Genotype	Mean	Std. deviation	Minimum	Maximum
Individual	WT L77	18.392	6.609	12.705	25.813
	WT Tor.	17.591	8.829	8.869	29.893
	RMS1	5.936	2.735	2.556	8.498
	RMS3	11.347	1.774	8.916	13.169
Social	WT L77	17.039	3.851	13.628	21.725
	WT Tor.	38.711	15.808	23.658	60.691
	RMS1	7.764	3.453	5.097	12.493
	RMS3	17.279	4.322	11.998	21.518

### 3.5. Morphological results

To further examine the response of these genotypes to social conditions, we decided to analyze the root surface area (RSA) as an index of the growth and architecture of the roots (Table 5).

**Table 5.** ANOVA test for the RSA on the considered plants.

Cases	Sum of squares	df	Mean square	F	p
Stimulus	2851.696	7	407.385	7.622	<.001
Residuals	1282.788	24	53.450		

Type III Sum of Squares.

**Figure 5.**
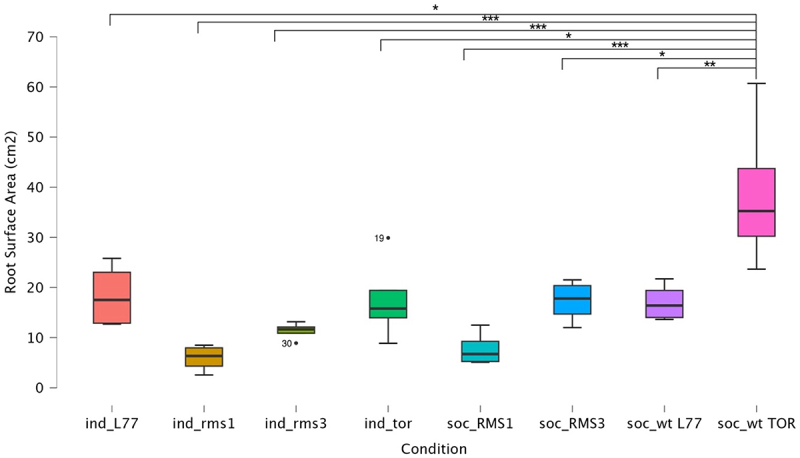
Box-plot for the root surface area (RSA) of all the genotypes in all the considered conditions. Significance is flagged as * *p* < .05, ** *p* < .01, *** *p* < .001.

When looking at the results for the RSA, the RSA of the wild-type Torsdag is significantly greater than the RSA of the other plants (see Tables 4, 5 and Figure 5) in both individual and social conditions (Figure 5). We found a significant difference in RSA between the individual and the social condition only for the Torsdag genotype (see Figure 5). For the social conditions, we found a significant difference between Torsdag and *rms3*, in which RSA was greater for the wild type, no significant differences were observed between L77 and *rms1* (see Figure 5).

## 4. Discussion

The aim of the present study was to investigate how strigolactones modulate a competitive situation among two pea plants via 3D motion analysis.^13,14^ The findings reveal distinct differences in ascent and attach behavior depending on the plant combination.

### 4.1. Comparing individual and social conditions for each genotype

Starting with the wild-type L77, a similar behavior for both the individual and social condition was found. It is important to recall that for the social condition, the wild-type L77 was paired with the *rms1–1* mutant. The results suggest that nesting the *rms1–1* mutant in the social condition does not influence the reach-to-grasp behavior of the wild-type L77. This observation can be explained by considering that the *rms1–1* mutant lacks in the ability to synthesize strigolactones (SLs). Consequently, the wild-type L77 may not detect the presence of a neighboring plant through SL emissions, leading to a behavioral pattern that remains consistent with that observed for the individual condition.

These findings become even more intriguing when we consider the differences in *rms1–1* behavior for the individual and the social conditions. From a kinematic standpoint, the *rms1–1* mutant exhibits a higher speed in the social compared to the individual condition. This result provides valuable insights for explaining the interaction occurring between the *rms1–1* mutant and the wild-type L77 in the social condition. It suggests that *rms1–1*, upon perceiving and reabsorbing SLs produced by the wild-type, increases its speed and modifies its behavior accordingly.

Turning to the wild-type Torsdag, its kinematic behavior differs for the individual and the social condition with the latter exhibiting a slower speed. It is essential to note that while the Torsdag wild-type successfully grasped the support in the individual condition, it failed to do so in the social condition when paired with the *rms3–1* mutant. Similarly, the *rms3–1* mutant displayed distinct behavioral patterns for the individual and social the condition, exhibiting a reduced speed for the social condition relative to the individual condition, mirroring the behavior observed for the wild-type Torsdag.

To recap, both the *rms1–1* and the *rms3–1* mutants failed to grasp the support in both the individual and the social conditions, though showing opposite velocity responses. The *rms1–1* mutant increased its velocity for the social condition compared to the individual condition, whereas the *rms3–1* mutant exhibited higher velocity for the individual condition compared to the social condition. In contrast, both the wild-type L77 and the wild-type Torsdag displayed similar grasping behavior for the individual condition, confirming the idea of a shared baseline behavior across the wild-type pea plants which have the ability of synthesizing and perceiving SLs.

### 4.2. Comparing rms1–1 mutants with wild type L77

Considering the combination of the *rms1–1* mutant, which does not synthesize SLs, and the wild type L77, we can recognize the pattern observed in Bonato et al.^13^ when two *Pisum Sativum* wild types were tested. This combination consists of a ‘winner’ plant that successfully grasped the support and a ‘loser’ plant that behaved in a very disoriented manner and was unable to implement the grasping phase. In the present study, the wild-type L77 could be considered the “winner” because it successfully grasped the support, while the *rms1–1* mutant could be considered the “loser” plant that moved cautiously, and it was unable to implement the necessary speed to compete with the other plant so as to accomplish the grasping phase.^13,24^ In general, the results indicate that mutant plants are unable to locate and grasp a potential support. Their movement appears to be disoriented and much less energized. These results confirm that specific mutations could lead to modifications at the behavioral level not only when mutants act in isolation,^15^ but also when nested in a competitive situation.

For the wild-type L77, a similar behavior for both the individual and social condition was found. The results suggest that nesting the *rms1–1* mutant in the social condition does not influence the reach-to-grasp behavior of the wild-type L77. This observation can be explained by considering that the *rms1–1* mutant lacks the ability to synthesize strigolactones (SLs). Consequently, the wild-type L77 may not detect the presence of a neighboring plant through SL exudation, leading to a behavioral pattern that remains consistent with that observed for the individual condition.

These findings become even more intriguing when we consider the differences in *rms1–1* behavior for the individual and the social conditions. From a kinematic standpoint, the *rms1–1* mutant exhibits a higher speed in the social compared to the individual condition. This suggests that *rms1–1*, upon detecting SLs exuded by the wild-type, increases its speed of circumnutation and modifies its behavior accordingly.

### 4.3. Comparing rms3–1 mutants with wild-type Torsdag

Turning to the wild-type Torsdag, its kinematic behavior differs dramatically for the individual and the social condition with the latter exhibiting a slower speed. It is essential to note that while the Torsdag wild-type successfully grasped the support in the individual condition, it failed to do so in the social condition when paired with the *rms3–1* mutant, which cannot perceive SLs. It also shows a dramatic increase in root growth in the social condition, compared to the individual condition. It is likely that these responses are caused by increased SL exudation from the *rms3–1* line. Strigolactone signaling mutants are unable to undergo feedback down-regulation of strigolactone synthesis, and as such produce and exude more strigolactones than wild-type.^12^ This excess of SLs in the soil may inhibit the ascent and attach behavior and cause a switch in competition from the shoot to the root. The Torsdag wild-type shows an exaggerated RSA with respect not only to the *rms3–1* mutant but also to the other genotypes (i.e., *rms1–1* and wildtype L77). The unsuccessful ascent behavior of the Torsdag wild-type toward the support could also potentially be attributed to the reallocation of energy predominantly to the below-ground structures rather than the aerial components responsible for grasping. This phenomenon aligns with the principles of functional equilibrium and the interactivity between plant modules, modulated by SLs both above and below ground^25–29^

The *rms3–1* mutant displayed distinct behavioral patterns for the individual and social condition, exhibiting a reduced speed for the social condition relative to the individual condition, mirroring the behavior observed for the wild-type Torsdag. This is intriguing when considering that *rms3–1* is unable to perceive SLs and therefore perceive its neighbors through this mechanism. It might be that instead the response of *rms3–1* is driven by the overgrowth of roots in its Torsdag neighbors. While both the *rms1–1* and the *rms3–1* mutants failed to grasp the support in both the individual and the social conditions, they showed opposite velocity responses in the social condition. The *rms1–1* mutant increased its velocity for the social condition compared to the individual condition, whereas the *rms3–1* mutant exhibited higher velocity for the individual condition compared to the social condition. In contrast, both the wild-type L77 and the wild-type Torsdag displayed similar grasping behavior for the individual condition, confirming the idea of a shared baseline behavior across the wild-type pea plants which have the ability to synthesize and perceive SLs.

## 5. Conclusions

In conclusion, this study provides foundational insights into the behavioral and physiological mechanisms governing plant interactions within social contexts. The ability to make “social decisions” in ecological settings may be mediated by SLs, with plants interpreting SL concentrations as part of an intricate signaling system. This research contributes to the broader understanding of plant communication and suggests that we may be progressively unraveling the complexities of this biochemical language.

## Supplementary Material

Supplemental Material

Supplementary material_Tables_bonato.docx

## Data Availability

The data that support the findings of this study are openly available in ZENODO at https://doi.org/10.5281/zenodo.15172295.
