# Supplementary figures and images for: Sizing up competition with strigolactones: the case of pea plants
Source: Plant Signal Behav. 2025 May 19;20(1):2506556. doi: 10.1080/15592324.2025.2506556 (PMC12091921; doi:10.1080/15592324.2025.2506556)

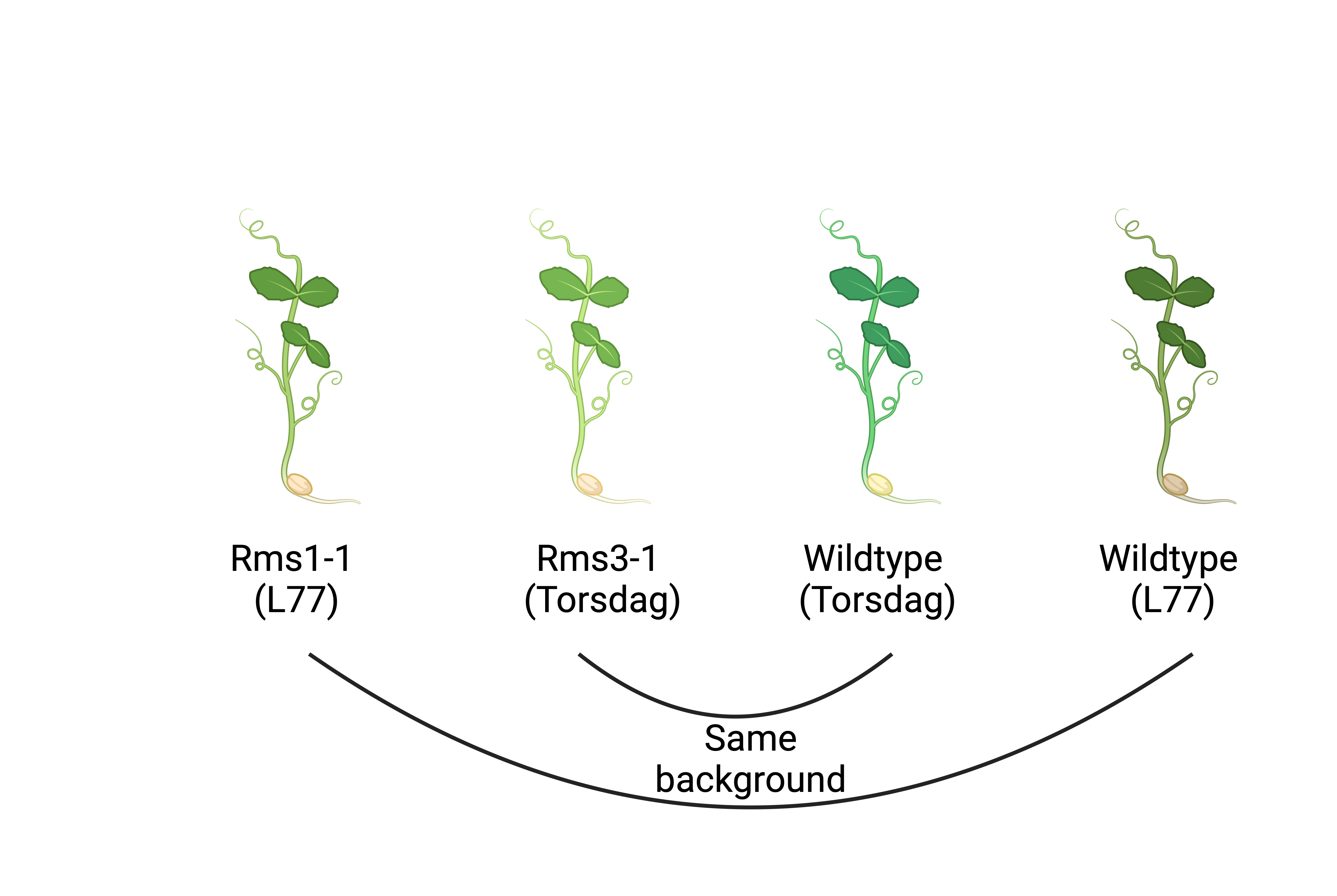

Supplement: Supplemental Material [file KPSB_A_2506556_SM7876.zip › Videos for Supplementary/figure 1.jpeg]

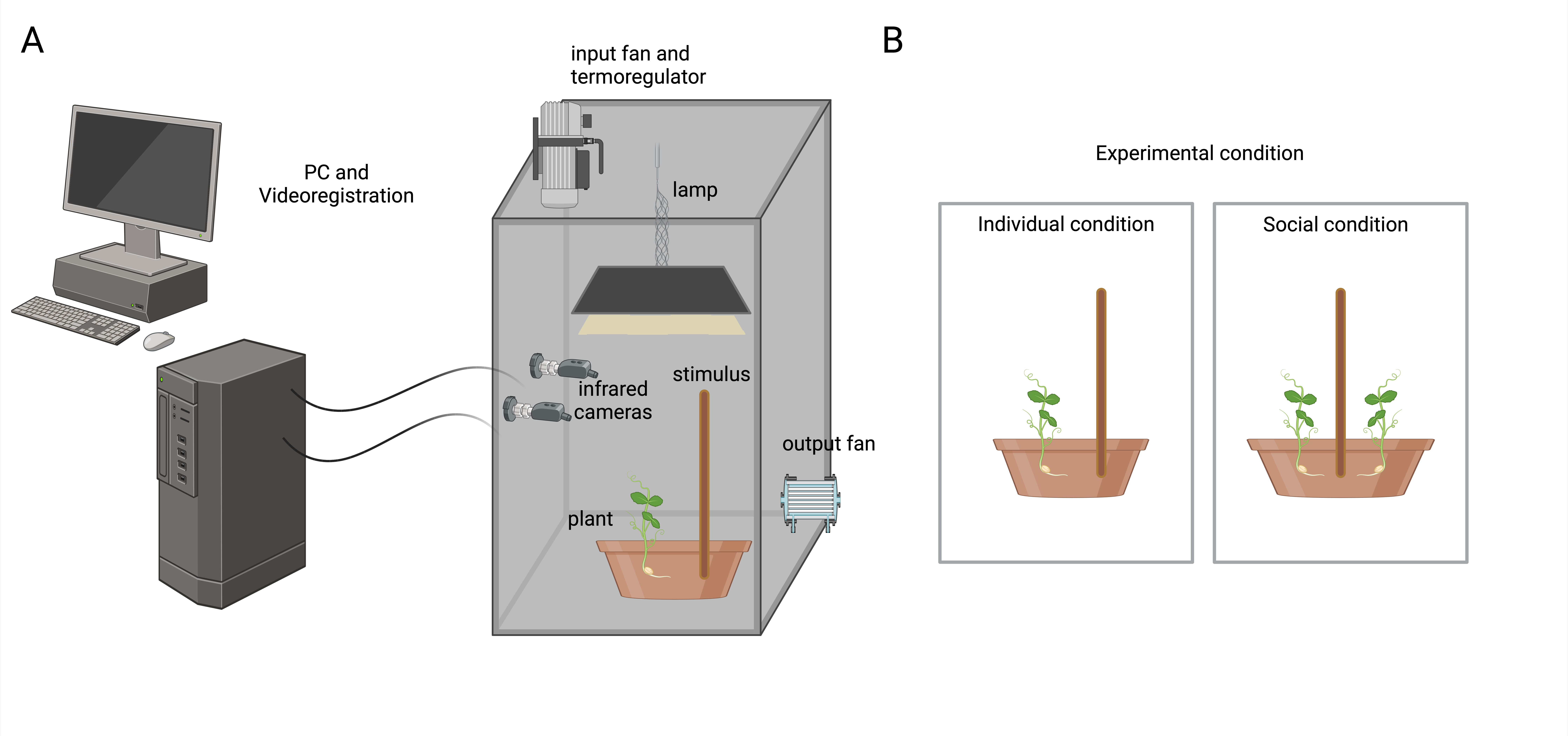

Supplement: Supplemental Material [file KPSB_A_2506556_SM7876.zip › Videos for Supplementary/figure 2.jpeg]

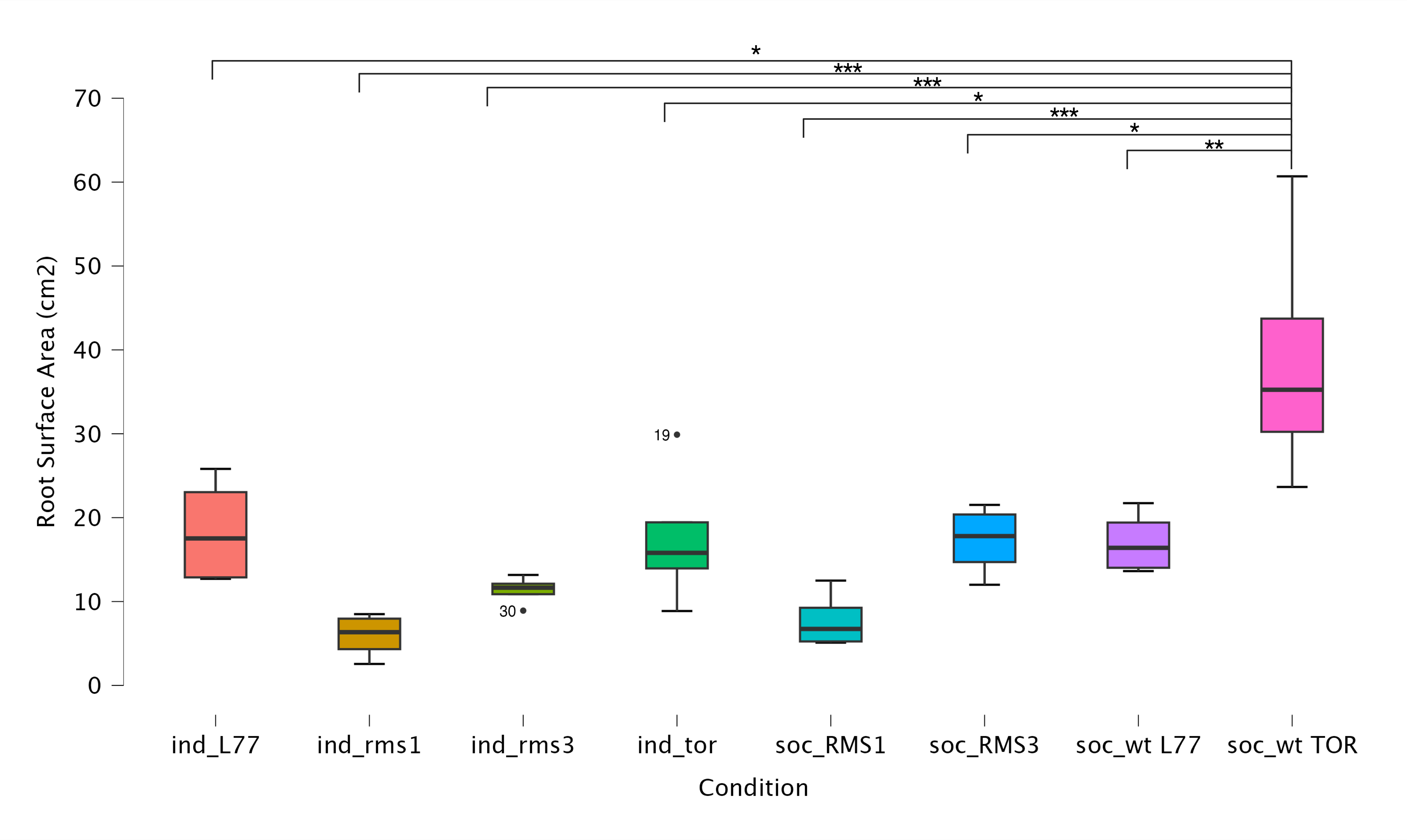

Supplement: Supplemental Material [file KPSB_A_2506556_SM7876.zip › Videos for Supplementary/figure 5.jpeg]
