# Supplementary material for: Sizing up competition with strigolactones: the case of pea plants
Source: Plant Signal Behav. 2025 May 19;20(1):2506556. doi: 10.1080/15592324.2025.2506556 (PMC12091921; doi:10.1080/15592324.2025.2506556)
Supplement: Supplementary material_Tables_bonato.docx [file KPSB_A_2506556_SM5598.docx]

**Supplementary material**

| Table S1. Bayesian Mann-Whitney U Test for the differences between the Wild-type L77 individual and social conditions. | | | | | | | |
| --- | --- | --- | --- | --- | --- | --- | --- |
|  | | **BF₁₀** | | **W** | | **Rhat** | |
| Number of circumnutations |  | 9.432 |  | 971.500 |  | 1.013 |  |
| Duration of circumnutations (min) |  | 1.311 |  | 1156.500 |  | 1.006 |  |
| Amplitude of mean velocity (mm/min) |  | 0.242 |  | 1702.000 |  | 1.019 |  |
| Distance from the support (mm) |  | 403.677 |  | 2312.000 |  | 1.051 |  |
|  | | | | | | | |
| *Note.*  Result based on data augmentation algorithm with 5 chains of 1000 iterations. | | | | | | | |

| Table S2. Bayesian Mann-Whitney U Test for the differences between the Wild-type Torsdag in individual and social conditions. | | | | | | | |
| --- | --- | --- | --- | --- | --- | --- | --- |
|  | | **BF₁₀** | | **W** | | **Rhat** | |
| Number of circumnutations |  | 0.670 |  | 2378.500 |  | 1.000 |  |
| Duration of circumnutations (min) |  | 0.856 |  | 1652.500 |  | 1.013 |  |
| Amplitude of mean velocity (mm/min) |  | 2.633×10^+7^ |  | 4051.000 |  | 1.007 |  |
| Distance from the support (mm) |  | 0.224 |  | 1447.000 |  | 1.022 |  |
|  | | | | | | | |
| *Note.*  Result based on data augmentation algorithm with 5 chains of 1000 iterations. | | | | | | | |

| Table S3. Bayesian Mann-Whitney U Test between the Rms1-1 mutant in individual and social conditions. | | | | | | | |
| --- | --- | --- | --- | --- | --- | --- | --- |
|  | | BF₁₀ | | W | | Rhat | |
| Number of circumnutations |  | 0.179 |  | 3969.000 |  | 1.005 |  |
| Duration of circumnutations (min) |  | 35.598 |  | 4949.500 |  | 1.024 |  |
| Amplitude of mean velocity (mm/min) |  | 16.958 |  | 2416.000 |  | 1.058 |  |
| Distance from the support (mm) |  | 0.336 |  | 2875.000 |  | 1.059 |  |
|  | | | | | | | |
| Note.  Result based on data augmentation algorithm with 5 chains of 1000 iterations. | | | | | | | |

| Table S4. Bayesian Mann-Whitney U Test between the Rms3-1 mutant in individual and social conditions. | | | | | | | |
| --- | --- | --- | --- | --- | --- | --- | --- |
|  | | BF₁₀ | | W | | Rhat | |
| Number of circumnutations |  | 0.371 |  | 2442.000 |  | 1.008 |  |
| Duration of circumnutations (min) |  | 43.199 |  | 1123.500 |  | 1.029 |  |
| Amplitude of mean velocity (mm/min) |  | 27.946 |  | 3105.000 |  | 1.029 |  |
| Distance from the support (mm) |  | 3847.743 |  | 618.000 |  | 1.106 |  |
|  | | | | | | | |
| Note.  Result based on data augmentation algorithm with 5 chains of 1000 iterations. | | | | | | | |
